# Triacylglycerols Composition and Volatile Compounds of Virgin Olive Oil from Chemlali Cultivar: Comparison among Different Planting Densities

**DOI:** 10.1100/2012/354019

**Published:** 2012-04-26

**Authors:** Mokhtar Guerfel, Mohamed Ben Mansour, Youssef Ouni, Flamini Guido, Dalenda Boujnah, Mokhtar Zarrouk

**Affiliations:** ^1^Institut Supérieur de Biologie Appliquée de Medenine, Université de Gabes, BP 522, 4100 Medenine, Tunisia; ^2^Laboratoire Caractérisation et Qualité de l'Huile d'Olive, Centre de Biotechnologie de Borj Cedria, BP 901, 2050 Hammam-Lif, Tunisia; ^3^Dipartimento di Scienze Farmaceutiche, Sede di Chimica Bioorganica e Biofarmacia, Via Bonanno 33, 56126 Pisa, Italy; ^4^Institut de l'Olivier, Station de Sousse, BP 14, 4061 Sousse, 4061 Sousse, Tunisia

## Abstract

The present study focused on the comparison the chemical composition of virgin olive oil samples obtained from fruits of the main Tunisian olive cultivar (Chemlali) grown in four planting densities (156, 100, 69, and 51 trees ha^−1^). Despite the variability in the triacylglycerols and volatile compounds composition, the quality indices (free fatty acids, peroxide value, and spectrophotometric indices K_232_ and K_270_) all of the virgin olive oils samples studied met the commercial standards. Decanal was the major constituent, accounting for about 30% of the whole volatiles. Moreover, the chemical composition of the volatile fraction of the oil from fruits of trees grown at the planting density of 156, 100, and 51 trees ha^−1^ was also characterised by the preeminence of 1-hexanol, while oils from fruits of trees grown at the planting density of 69 trees ha^−1^ had higher content of (*E*)-2-hexenal (20.3%). Our results confirm that planting density is a crucial parameter that may influence the quality of olive oils.

## 1. Introduction

Virgin olive oil composition determines its intrinsic quality and could be influenced by several factors. Cultivar, environment, and agronomic practices affect the fruit physiology, whereas processing and storage conditions alter oil composition. Virgin olive oil is composed of triacylglycerols (around 97-98%), minor variable amounts of free fatty acids, and minor glyceridic compounds—partial glycerides, phospholipids, and oxidized triacylglycerols—and around 1% of unsaponifiable constituents of varied structure and polarity [1]. Virgin olive oil is characterized by a unique flavour, which represents one of the most important qualitative aspects of this vegetable oil, and plays a major role in consumer approval. Although a full description of the organoleptic characteristics of the oil is only obtainable through sensory analysis, the quali-quantitative determination of the volatile compounds can provide very useful information on product quality. The volatile composition of olive oil includes aldehydes, ketones, alcohols, and esters [2]. Furthermore, it is well established that aliphatic C6 compounds (aldehydes, alcohols, and their corresponding esters) are the most abundant compounds of virgin olive oil aroma [3, 4]. They mainly contribute to its green odour notes [5, 6]. The study of volatile compounds has been successfully used for the quality control of olive oils, particularly for the detection of adulterants [7] or rancidity (oxidation) [8] or to determine their origin [7].

Tunisia is a very important country in the olive oil producing world, the largest African exporter and fourth worldwide after Spain, Italy, and Greece [9]. The olive tree (*Olea europaea* L.) is present practically in every region of the country, up to the border of the southern desert.

Historically, Tunisian olives were produced under dry-land conditions where trees were spaced widely to take full advantage of the stored soil water from winter rains for spring and summer growth. Many studies have been carried out to categorise virgin olive oils from different cultivars and different geographical origins according to their different chemical properties. However, there is no study on the effect of the planting density on the volatile and triacylglycerols composition of olive oil. The present research aimed to study the relationships between the aroma profile and triacylglycerols composition of virgin olive and the planting density. For these purposes, the volatile compounds and triacylglycerols composition of virgin olive oils were compared in samples obtained from fruits of olive trees cultivated in four planting densities ranging from 51 to 156 tree ha^−1^.

## 2. Materials and Methods

### 2.1. Oil Sample Extraction

Olive oil samples were obtained from fruits of the main Tunisian olive cultivar, Chemlali, which were picked by hand at the same stage of maturity from three trees during the crop season 2009/2010 (October) in a 4 ha olive orchard located in Souassi center of Tunisia (35°.49′N, 10°.30′E). The climate of the study area is Mediterranean with an average annual rainfall of 250 mm, mostly distributed outside a 4-month summer drought period. Olive samples were collected from trees planted in 1988, at four-tree spacing: 8 × 8 m, 10 × 10 m, 12 × 12 m, and 14 × 14 m corresponding to 156, 100, 69, and 51 trees ha^−1^, respectively, and were subjected to the same fertilization and common olive cultivation practices. Tree heights were about 3 m. Higher trees productions were about 1300 kg ha^−1^. The same laboratory mill was used to prepare the olive oil samples. Only healthy fruits, without any kind of infection or physical damage, were processed. After harvesting, fresh olives (1.5–2.0 kg) were washed and deleafed, crushed with a hammer crusher, and the paste mixed at 25°C for 30 min, centrifuged without addition of warm water (oil produced from each extraction was 200–250 mL/kg) and then transferred into dark glass bottles, and stored (one week) in the dark at 4°C until analysis.

### 2.2. Determination of Oil Quality Parameters

Free acidity, expressed as percent of oleic acid (%18 : 1); peroxide value, given as milliequivalents of active oxygen per kilogram of oil (meq O_2_/kg); and UV absorption characteristics (K_232_ and K_270_) were determined according to the analytical methods described in the European Union Commission Regulations EEC/2568/91 and EEC/1429/92. 

### 2.3. Chromatographic Analysis of Triglycerides

A 0.3 g oil sample was dissolved in 10 mL of 2-propanol/acetonitrile/n-hexane (2 : 2 : 1; v/v) and homogenized by stirring. High-performance liquid chromatography analyses were performed using an HP 1100 Series instrument (Agilent Technologies, Palo Alto, CA, USA) equipped with a binary pump delivery system, degasser, and autosampler and an evaporative light scattering detector (ELSD) PL-ELS model 1000 Series (Polymer laboratories, Varian Inc., Amherst, MA, USA). Luna C18 (Phenomenex, Torrance, CA, USA) column, 5 *μ*m particle size, 25 cm × 3.00 mm ID, with a C18 precolumn filter (Phenomenex) was used at room temperature. All solvents were filtered through a 0.45 *μ*m nylon filter disk (Lida Manufacturing Corp., Kenosha, WI, USA) prior to use. The injection volume was 10 *μ*L. The eluent used was a gradient of 2-propanol as mobile phase A, and acetonitrile as mobile phase B of the solvent system. Elution was performed at a solvent flow rate of 0.7 mL min^−1^ with a linear gradient as follows: from 0 to 2 min 52% B, up to 4 min 58% B, 25 min 58% B, 30 min 10% B, and 37 min 52% B. Peak assignment was carried out by comparison with chromatograms reported in the literature [10] and with several pure standards. The effluent was monitored with an evaporative light scattering detector, with the following settings: evaporator temperature 70°C; nebulizer, 30°C; transfer line, 30; and gas flow rate, 1.0 L min^−1^. 

### 2.4. Volatile Compound Analyses

Solid-phase microextraction devices coated with polydimethylsiloxane were used to sampling the headspace of 2 mL of olive oil inserted into a 5 mL glass septum vial and allowed to equilibrate for 30 min. After the equilibration time, the fibre was exposed to the headspace for 50 min at 25°C room temperature. Extraction temperature and times were optimised in previous studies. Once sampling was finished, the fibre was withdrawn into the needle and transferred to the injection port on the GC-FID and GC-MS system. GC-EIMS separations were performed with a Varian CP 3800 gas chromatograph equipped with a DB-5 Capillary column (30 m × 0.25 mm; coating thickness = 0.25 mm) and a Varian Saturn 2000 ion trap mass detector. Analytical conditions were as follows: injector and transfer line temperature at 250 and 240°C, respectively; oven temperature programmed from 60 to 240°C at 3°C min^−1^; carrier gas, helium at 1 mL min^−1^; and splitless injection. Identification of the constituents was based on comparison of the retention times with those of authentic samples, comparing their linear retention indices relative to the series of n-hydrocarbons, and on computer matching against commercial (NIST 98 and ADAMS) and homemade library mass spectra built from pure substances and components of known oils and MS literature data [11–14]. Moreover, the molecular weights of all the identification substances were confirmed by GC-CIMS, using MeOH as CI ionising gas. 

### 2.5. Statistical Analysis

Significant differences between means were determined by an analysis of variance, which applied a Duncan's test. Differences were considered statistically significant when the probability was greater than 99% (*P* < 0.01). The statistical analysis was performed using SPSS 13.0 for Windows (SPSS Inc., 2004). 

## 3. Results and Discussion

### 3.1. Oil Quality Parameters

Table 1 lists the mean and the standard deviation values of the most interesting analytical parameters of olive oil samples made out of the different planting density grown in the area of study (free fatty acid content, peroxide value, and extinction coefficients at 232 and 270 nm). 

All oils produced and analyzed showed very low values for the regulated physicochemical analytical parameters evaluated, with all of them falling within the “extra virgin” category, as stated by Regulation EC/1989/2003. This is not surprising since the raw material was carefully selected, picked, and processed. Note that lower values for these parameters will be translated into a higher quality of oil. These results are consistent with the findings of [15, 16]. In fact, they reported that these analytical parameters are basically affected by factors causing damage to the fruits (e.g., olive fly attacks or improper systems of harvesting, transport, and storage of olives). 

### 3.2. Triacylglycerols

Results for triacylglycerol contents, expressed in percentage of total triacylglycerols of oil samples, are shown in Table 2. The analysis of triacylglycerols permitted the identification and the quantification of 10 triacylglycerols (TAGs). PLL, OLL, PPO, SOO, and POLn + EeOL were present in low percentages, whereas OOL, POL, OOO, and POO accounted for more than 85% of the total area of peaks in the chromatographic profile. Triacylglycerol contents, expressed in percentage of total triacylglycerols (Table 2), showed variations between samples from different planting densities. In relation to the main TAGs (OOL, POL, OOO, and POO), the level of triolein (OOO) was remarkably high, ranging from 28.5% to 31.7%. Among the studied samples, olive oil samples obtained from fruits of trees grown at the planting density of 51 trees ha^−1^ registered the lowest percentages of triolein. The second peak in order of quantitative importance in studied virgin olive oils corresponded to the POO which ranged from 25.8% to 30.30%. Olive oil samples obtained from fruits of trees grown at the planting density of 51 trees ha^−1^ had higher amounts of POL (16.4%) and OOL (17.50%). As for fatty acids [17], the composition of triacylglycerols of Chemlali oils showed variations depending on the planting density. 

### 3.3. Volatile Compounds Analyses

Aroma is an important criterion for virgin olive oils. Volatile components of olive oil are of great interest since they are related to its quality and are used to detect adulteration [18]. All the identified volatiles are listed in Table 3. Several compounds have been characterised by GC-FID and GC-MS analysis. Decanal was the major constituent, accounting for about 20% of the whole volatiles (Table 3). Other compounds present in relatively high concentrations were 1-hexanol, (*E*)-2-hexenal,ortho-guaiacol, (*E*)-**β**-ocimene, phenyl ethyl alcohol, and (*E*,*E*)-*α*-farnesene. As shown in Table 3, the chemical composition of the volatile fraction of Chemlali olive oils varies widely, depending on the planting density. A direct comparison of our results in this study with the literature data is not possible because of the great variability of the volatile composition with reference to the different ripeness stages of olives, extraction techniques, and analytical methods. According to Kiritsakis et al. [16] and Salas and Sanchez [19], methods used and conditions applied to obtain olive oil from olive fruit affect its volatile composition. In fact, the number of volatile compounds detected in the aroma of an olive oil depends on the quality of the virgin olive oil and on the methodology adopted for their determination. Thus, depending on the temperature and time used for obtaining the volatile fraction, different results can be found [20].

Several terpene hydrocarbons (mono- and sesquiterpenes) were often detected, and they totally accounted for 0–15.3% of the whole volatiles (Table 3). (*E*,*E*)-*α*-farnesene (1.2–15.3%) was the main one. The hydrocarbons of olive oil have been studied by different authors as possible markers to distinguish virgin olive oil from different olive varieties or geographical origins [21–24]. The volatile fraction of the oil from fruits of trees grown at the planting density of 156 trees ha^−1^ was characterised by the preeminence of two compounds: 1-hexanol (15.1%) and decanal (37.4%). The other main compounds detected were 1-hexyl acetate (11.8%), (E)-2-hexenal (7.4%), and phenyl ethyl alcohol (7.7%) (Table 3). Therefore, the chemical composition of the volatile fraction of the oil from fruits of trees grown at the planting density of 100 trees ha^−1^ was characterised by the preeminence 1-hexanol (32%), Decanal (39%), (*E*)-2-hexenal (10.9%), and (*E*)-**β**-ocimene (4.1%) (Table 3). Other minor volatile compounds were observed in the virgin olive oils: *β*-caryophyllene, valencene, (*E*,*E*)-*α*-farnesene, (*E*)-2-nonenal, and benzyl alcohol. The major constituents of the volatile fraction obtained from of the oil from fruits of trees grown at the planting density of 69 trees ha^−1^ were identified as decanal (28.6%), (*E*)-2-hexenal (20.3%), and 1-heptadecene (15.4%). Other important volatiles were 1-hexanol (7.3%), Hexanal (4.4%), and pentadecane (2.8%) (Table 3). Also, the volatile fraction of oil from fruits of trees grown at the planting density of 51 trees ha^−1^ oil was characterised by the preeminence of Decanal (28.8%), 1-hexanol (19.3%), (*E*,*E*)-*α*-farnesene (15.3%), and ortho-guaiacol (10.1%). Other minor volatile compounds were observed in the virgin olive oils (Table 2): (*E*)-**β**-ocimene (4.7%), 2,3-butanediol (3.3%) 2-ethylbenzaldehyde (2.2%), and (*Z*)-3-hexen-1-ol (3%). Therefore, the differences between the four planting densities were mainly quantitative because most of the compounds were detected in all the olive oils. The variation in levels of C6 aldehydes and alcohols for oil samples from different regions implies that planting density may influence the activity of Alcohol Dehydrogenase (ADH). Differences in the levels of esters in olive oil were also observed between olive oil samples, suggesting a dependence of Alcohol Acetyl Transferase (AAT) activity on planting density. 

Comparing the four olive oil samples collected at full maturity and grown under the same conditions, we can observe that there were differences in the composition of volatile fraction. In addition to the ones listed previous, it can also be mentioned that 1-heptadecene was detected only in the volatile fraction of oil from fruits of trees grown at the planting density of trees ha^−1^ (Table 3). Since the volatile compounds influencing the taste and aroma of the virgin olive oils samples are formed in the olive fruit through enzymatic processes, they are affected by the olive cultivar, the origin, the maturity stage of the fruit at the time point of collection, and the olive fruit processing. The enzymatic oxidation of linoleic and linolenic acids by the lipoxygenase cascade (LOX) gives rise to the major compounds of the volatile fraction in good quality virgin olive oils [25]. In the studied olive oils samples, the C6 and C5 compounds were the main fractions, except for oil from fruits of trees grown at the planting density of 100 trees ha^−1^ that had high concentrations of alcohols. These results may be explained by a different activity of the enzyme alcohol dehydrogenase, which reduces the C6 aldehydic compounds to the corresponding alcohols. 

## 4. Conclusions

In conclusion, despite that, olive oil samples were for all planting densities within the limits established in the European Regulation, allowing them to be classified as extra virgin olive oils. Olive oil samples obtained from fruits of trees grown at the four planting density were found to have varied volatiles compound contents and triacylglycerols composition. Olive trees planted closer together compete with their neighbours for growing space and resources. Moreover, our results confirm that planting density is a crucial parameter similar to other factors (olive cultivar, the origin, the maturity stage of the fruit at the time point of collection, and the olive fruit processing) that may influence the quality of olive oils. 

## Figures and Tables

**Table 1:** Quality parameters of Chemlali olive oil samples from the four planting densities.

	156 trees ha^−1^	100 trees ha^−1^	69 trees ha^−1^	51 trees ha^−1^
Acidity (%C18 : 1)	0.60 ± 0.06b	0.72 ± 0.10c	0.62 ± 0.03b	0.48 ± 0.12a
PV (meq O_2_/Kg oil)	4.0 ± 2.00a	5.0 ± 1.00a	6.0 ± 1.15a	8.0 ± 0.58b
^ K^232	2.40 ± 0.18a	2.50 ± 0.09a	2.10 ± 0.07a	2.32 ± 0.08a
^ K^270	0.21 ± 0.01b	0.23 ± 0.09b	0.13 ± 0.08a	0.13 ± 0.01a

^a–d^Mean ± SD, significant differences within the same row are shown by different letters (*P* < 0.001).

PV: peroxide value; K_232_ and K_270_: values of specific extinction given as absorbance at 232 and 270 nm, respectively.

**Table 2:** Triacylglycerol composition of the studied olive oils.

	156 trees ha^−1^	100 trees ha^−1^	69 trees ha^−1^	51 trees ha^−1^
PLL	0.00a	0.00a	0.00a	0.21a
OLL	0.50b	0.20a	0.20a	0.38b
POLn + EeOL	0.36a	2.44b	3.33c	3.26c
OOL	9.85a	12.80b	13.50b	17.55c
POL	3.50a	10.80b	11.67b	16.40c
OOO	29.42a	30.04b	31.77c	28.57a
POO	30.95a	35.34b	34.42b	30.87a
PPO	0.67a	2.29b	2.34b	2.22b
SOO	1.10c	0.67b	0.40a	0.30a

Significant differences within the same row are shown by different letters. OLL: 1-oleyl-2-linoleyl-3-linolenoylglycerol; OOL: 1,2-dioleyl-3-linolenoylglycerol; PLL: 2,3-dilinoleyl-1palmitoylglycerol; OOL: 1,2-dioleyl-3-linoleyglycerol; POL: -palmitoyl-2-oleyl-3-linoleylglycerol; OOO: 1,2,3-trioleylglycerol; POO: 2,3-dioleyl-1-palmitoylglycerol; SOO: 2,3-dioleyl-1stearoylglycerol.

**Table 3:** Composition of the volatile fraction obtained from Chemlali virgin olive oils extracted by HS-SPME.

	LRI	156 trees ha^−1^	100 trees ha^−1^	69 trees ha^−1^	51 trees ha^−1^
2,3-butanediol	792	—	—	—	3.3a
Hexanal	800	2.7b	3.8c	4.4c	0.9a
(*E*)-2-hexenal	851	7.4a	10.9b	20.3c	—
(*Z*)-3-hexen-1-ol	853	—	—	—	3.0a
1-hexanol	871	15.1b	32.0d	7.3a	19.3c
propyl butanoate	902	—	—	1.6	—
(*E*,*E*)-2,4-hexadienal	912	—	—	—	—
propyl 2-methylbutanoate	953	—	—	1a	—
(*Z*)-2-heptenal	962	—	—	—	—
1-heptanol	970	—	—	—	0.2a
Phenol	982	—	—	—	1.1a
3-octanone	987	—	—	0.4a	0.3a
(*E*,*Z*)-2,4-heptadienal	998	—	—	—	—
(*Z*)-3-hexenyl acetate	1007	4.5	—	—	—
1-hexyl acetate	1009	11.8b	—	—	0.3a
pentyl propanoate	1012	—	—	2.1	—
benzyl alcohol	1033	0.6a	0.3a	—	1.9
(*E*)-**β**-ocimene	1051	0.5a	4.1b	1a	4.7b
pentyl isobutyrate	1058	—	—	0.9a	—
ortho-guaiacol	1090	—	—	—	10.1a
isopentyl 2-methylbutanoate	1099	—	—	0.5a	—
isopentyl isovalerate	1103	—	—	1.6a	—
nonanal	1104	0.8a	0.6a	—	0.5a
phenyl ethyl alcohol	1112	7.7b	—	1.3a	1.3a
veratrole	1148	—	—	—	0.2a
*p*-vinyl anisole	1155	—	0.4a	—	—
(*E*)-2-nonenal	1164	—	0.6a	—	1a
Decanal	1206	37.4a	39.9a	28.6a	28.8a
2-ethylbenzaldehyde	1219	—	—	—	2.2a
Tridecane	1300	—	—	—	—
*α*-copaene	1377	—	—	—	—
1-tetradecene	1390	—	—	1.0a	—
Isocaryophyllene	1407		0.1a	—	—
**β**-caryophyllene	1418	1.3a	0.7a	—	—
dihydro-b-ionone	1435	—	—	—	—
*α*-humulene	1456	0.7a	—	0.1a	—
*γ*-muurolene	1477	—	—	—	—
valencene	1494	1.0a	1.2a	—	0.6a
pentadecane	1500	—	—	2.8a	—
(*E,E*)-*α*-farnesene	1505	2.5b	1.2a	2.3b	15.3a
Cedrol	1597	—	—	—	—
1-heptadecene	1678	—	—	15.4a	—
heptadecane	1700	—	—	1.1a	—
Total identified		94.5	97.2	92.3	95.0
